# Regulatory T Cells-Related Genes Are under DNA Methylation Influence

**DOI:** 10.3390/ijms22137144

**Published:** 2021-07-01

**Authors:** Magdalena Piotrowska, Mateusz Gliwiński, Piotr Trzonkowski, Dorota Iwaszkiewicz-Grzes

**Affiliations:** Department of Medical Immunology, Medical University of Gdansk, 80-210 Gdańsk, Poland; m.piotrowska@gumed.edu.pl (M.P.); mateusz.gliwinski@gumed.edu.pl (M.G.); ptrzon@gumed.edu.pl (P.T.)

**Keywords:** Tregs, Tconv, DNA methylation, gene expression, epigenetic modifiers

## Abstract

Regulatory T cells (Tregs) exert a highly suppressive function in the immune system. Disturbances in their function predispose an individual to autoimmune dysregulation, with a predominance of the pro-inflammatory environment. Besides *Foxp3*, which is a master regulator of these cells, other genes (e.g., *Il2ra, Ctla4, Tnfrsf18, Ikzf2,* and *Ikzf4*) are also involved in Tregs development and function. Multidimensional Tregs suppression is determined by factors that are believed to be crucial in the action of Tregs-related genes. Among them, epigenetic changes, such as DNA methylation, tend to be widely studied over the past few years. DNA methylation acts as a repressive mark, leading to diminished gene expression. Given the role of increased CpG methylation upon Tregs imprinting and functional stability, alterations in the methylation pattern can cause an imbalance in the immune response. Due to the fact that epigenetic changes can be reversible, so-called epigenetic modifiers are broadly used in order to improve Tregs performance. In this review, we place emphasis on the role of DNA methylation of the genes that are key regulators of Tregs function. We also discuss disease settings that have an impact on the methylation status of Tregs and systematize the usefulness of epigenetic drugs as factors able to influence Tregs functions.

## 1. Introduction

Regulatory T cells (Tregs) are CD4^+^ suppressor cells that have the capacity to block T effector cells (Teffs). The majority of Tregs-related examinations have confirmed their ability to mediate specific immune system inhibition and their usefulness in many clinical trials. Tregs, as a drug, are used in the treatment of autoimmune diseases and conversely their depletion is considered as a promising tool for cancer patients. Conducting research on regulatory T cells has revealed a great deal of factors that can affect their stability and phenotype, which might be the next step to develop Tregs-related therapy.

Tregs are a heterogeneous population of CD4-positive T cells determined by high expression of CD25 and low expression of CD127. The protein Foxp3 is considered to be the major transcription factor responsible for Tregs function and stability. Besides *Foxp3*, there are many other Tregs-signature genes, coding factors such as *Il2ra* (CD25), *Ctla4* (CD152), *Tnfrsf18* (GITR), *Ikzf2* (Helios), and *Ikzf4* (Eos), which are believed to play an important role in Tregs function [1,2,3,4]. Foxp3-positive T cells can be divided into different subpopulations according to their origin: best characterized as, and believed to be highly effective and stable, thymus-derived Tregs (nTregs), peripheral-induced Tregs (pTregs), and in-vitro-generated (iTregs), produced from CD4^+^CD25^−^ naïve T cells. Another division according to phenotype diversity, due to Tregs functional state, allows to distinguish CD44^low^CD62L^high^ (central, cTregs) and CD44^high^CD62L^low^ (effector, eTregs). Natural and induced Tregs are characterized by the factors Helios (an Ikaros transcription factor) and Neuropilin-1, which are thought to be specific for Tregs that arose in the thymus [5,6,7].

Epigenetics describe changes in gene expression not caused by alterations in the DNA nucleotide sequence. The most important and well known are DNA methylation, histone posttranslational modifications, and inhibition by non-coding RNA (ncRNA). Each epigenetic mark is responsible for maintenance of DNA availability and results in changes in gene expression as well as in chromatin structure. Subsequent chromatin remodeling has an impact on the production of many crucial proteins for the proper action of the immune system [8,9].

DNA methylation is a process of cytosine conversion into 5-methylocitosine catalyzed by specific enzymes called DNA methyltransferases (DNMTs), which use S-adenosylmethionine as a substrate. DNMTs are a group of enzymes—DNMT3A, DNMT3B, and DNMT1—that transfer the methyl group during de novo methylation or during cell replication. This reaction is presented at CpG islands, highly enriched in CG content. When CpG islands are methylated, it is believed that gene expression is silenced, and the gene is repressed. TET enzymes (Ten-Eleven Translocation family enzymes) are able to reverse the process and induce a demethylation process that leads to an open chromatin structure, and eventually to gene expression [10,11].

In the case of regulatory T cells, DNA methylation is particularly important. Several studies revealed more than 100 differentially methylated regions (DMRs) in Tregs compared to Teffs. CpG hypomethylation in Tregs-related genes, such as *Ctla4* exon 2, *Foxp3* intron 1, *Tnfrsf18* exon 5, *Ikzf4* intron 1b, and *Il2ra* intron 1a, is limited to Tregs and persists after cell stimulation [12,13]. It is worth emphasizing that Tregs-specific hypomethylation of critical genes is a dynamic process during Tregs development. It begins at the precursor stage and continues until full cellular maturity [14].

**Figure 1:**
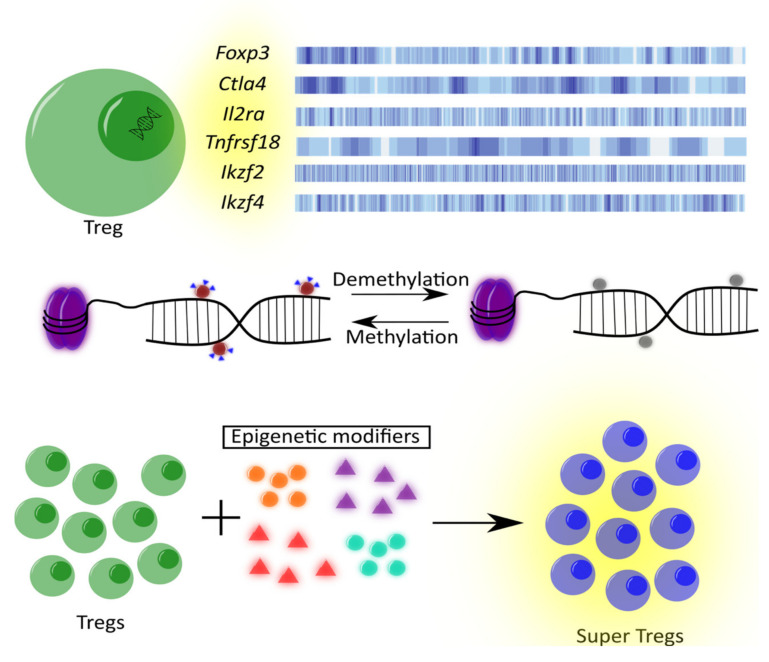
The main purpose of the article is shown. Genes that are under discussion are presented as well as the role of DNA methylation and epigenetic modifiers in regulatory T cells.

In this review, we discuss DNA methylation patterns in genes crucial for regulatory T cells, such as *Foxp3*, *Ctla4*, *Il2ra*, *Tnfrsf18*, *Ikzf2*, and *Ikzf4*. Moreover, we will give a brief overview on the role of DNA methylation in specific gene regions and its diversity in Tregs function and stability. The main objectives are presented in Figure 1.

## 2. DNA Methylation Overview

DNA methylation is a post-replicative process occurring in CpG islands in mammalian cells that is passed onto daughter cells and provides cell memory [1,2]. The definition of CpG islands said that these 200 bp regions comprise over 50% CG nucleotides. However, Takai and Jones proposed new criteria that require at least a 55% CG content and extended the length to 500 bp [15,16]. While disturbances in DNA methylation patterns predispose to the development and progression of cancer, it is highly important to preserve proper mechanisms allowing DNA methylation [3,4]. DNA methylation occurs with the presence of DNMTs having capacity to transfer a donor group (S-adenosyl-L-methionine) into the fifth carbon of cytosine residues in DNA. Such enzymes are divided into subgroups depending on their mechanism of action [17]. The first—DNA methyltransferase 1 (DNMT1)—prefers hemi-methylated DNA and enables it to maintain DNA methylation during cell divisions. The major function of DNMT1 was confirmed in the study of inheritance neurodegeneration, where mutations in the DNM1 gene caused hereditary sensory and autonomic neuropathy type 1 disease (HSAN1) [18,19]. On the other hand, DNMT3 isoforms are responsible for de novo methylation and consist of the catalytically active DNMT3A and DNMT3B and the inactive DNMT3L. During development and cell proliferation, mutations in DNMT3B contributes to immunodeficiency–centromeric instability–facial anomalies syndrome (ICF syndrome), which is an immunodeficiency disease with the presence of centromeric instability and facial anomalies. While mutations in DNMT3A act as negative prognostic factor in patients with acute myeloid leukemia (AML) [20,21], DNTM1 ablation in Teffs and its inhibition by DNMTs inhibitor 5-aza-2′-deoxycytidine (Aza) lead to an increase in *Foxp3* expression in these cells. On the contrary, deletion of DNMT1 in developing thymic Tregs results in lethal autoimmunity [8,22,23]. Moreover, the study on Uhrf1 proteins, which recruits DNMT1 and promotes DNA methylation in Tregs, revealed that mature Tregs lacking Uhrf1 have proinflammatory capacities [24].

The DNA demethylation process occurs in a passive and active manner. Passive global DNA demethylation due to cell proliferation is caused by insufficient inheritance of the DNA methylation pattern that can be restored by DNMT1 [25]. Active DNA demethylation is mediated by the three mammalian members of the TET family enzymes that are able to convert 5 mC into 5 hmC (5-hydroxymethylcytosine), 5 fC (5-formylcytosine), and 5 caC (5-carboxylocytosine) [26]. Elevated TET enzyme levels from low oxygen conditions promotes CNS2 demethylation and *Foxp3* stability [27]. Reports have shown that DNA methylation is a repressive mark and switches off the transcriptional machinery [28]. DNA methyltransferases, as was discussed above, play a crucial role in such mechanisms. Unfortunately, subsequent silencing of gene expression is a multidimensional process. A group of proteins (MeCP1-2, MBD1-4) possessing domains for methylated DNA (MBD—methyl-CpG-binding domain) are connected with gene repression owing to their capacity to recruit histone protein-modifying enzymes and chromatin remodeling. For example, MeCP2 silences gene expression through recruitment of histone deacetylase (HDAC) and allows lysine 9 in H3 methylation. Moreover, MeCP2 interacts with HP1 (heterochromatin protein 1), together leading to gene repression [15,16,29]. Furthermore, many transcription factors (TFs) are unable to bind to their ligands when methylated. For example, binding of reduced NFAT (Nuclear Factor of Activated T cells) in the *Ctla4* promoter was diminished due to enrichment in methylation level, suggesting a higher binding affinity for demethylated regions. In another study on NRF1 (Nuclear Respiratory Factor 1), it was also confirmed that this TF prefers binding to unmethylated promoters [30,31].

## 3. *Foxp3* Gene

### 3.1. Foxp3 Gene Structure

Human *Foxp3* gene is located on the X chromosome (Xp11.23). Its transcript contains 2264 bps and encodes 431 amino acids [32]. It consists of 12 exons (11 coding and 1 non-coding). Moreover, the 5′ part of exon 2 and 3′ part of exon 12 represent non-coding fragments. Mantel et al. using 5′-PCR RACE, have reported that the promoter region appears upstream approximately −6 kbp from the translation start site, and is conserved in humans, mice, and rats. They have also revealed the basal promoter targeting cell specificity, which is placed within the first 500 bp and is bound to AP-1 and NFAT transcription factors [33]. Typical *cis*-regulatory DNA components, such as enhancers, play a pivotal role in *Foxp3* regulation. Analysis of ~30 kb of mouse genomic DNA revealed an upstream enhancer −4970 to −6021 bp from the transcriptional start site (TSS) and confirmed its 92% conservativeness with human DNA [8]. Recent studies have revealed that analogous human upstream enhancer is about 800 bp and is placed approx. −6100 to −5300 bp from the TSS [34]. Another one, named CNS0 (conserved non-coding sequence), is placed ~8000 bp upstream of the TSS. This sequence is considered as a super-enhancer (SE), highly enriched in a massive amount of TFs that plays a pivotal role in gene expression, controlling the cell identity and fate [35]. Besides upstream promoter enhancers, there are downstream regulatory elements, located in intronic DNA sequences, referred to as CNS1-3 (according to their distance from TSS). CNS1 and CNS2 are located in the intronic sequence 1, while the CNS3 is placed in intron 2, between exon 1 and exon 2 [36].

### 3.2. Foxp3 DNA Methylation

As mentioned, methylation occurs in CpGs, which are included in *cis*-regulatory elements such as enhancers and promoters. The master regulator of Tregs is *Foxp3*, and its regulation is crucial for maintaining immune homeostasis. However, *Foxp3* expression alone is not sufficient to achieve full Tregs suppressive function and its coaction with CpG hypomethylation is required for Treg cells development. Okhura et al. confirmed that *Foxp3* expression alone was not enough for Tregs lineage generation and the presence of a genome-wide nTregs cell-type CpG hypomethylation pattern was crucial, and depended on T cell receptor (TCR) signaling [12].

The newly discovered CNS0 acts as an SE and is bound by a pioneer factor Satb1, triggering Tregs differentiation due to *Foxp3* induction. Disruption of Satb1-mediated SE activation not only leads to impaired *Foxp3* induction but also diminishes DNA demethylation in Tregs-specific demethylation regions (TSDRs) [35]. This region is also bound by STAT5, and cooperatively with Il-2 enables *Foxp3* induction in Tregs precursors, while its depletion together with *Aire* resulted in a worsened autoimmune disease [37]. Moreover, both naïve and activated Tregs comprise the Tregs-SEs, which correlate with Tregs-DRs (demethylated regions), affecting gene expression. What is more, autoimmune-disease-associated SNPs (single nucleotide polymorphisms), which affect Tregs function, are abundant in Treg-specific demethylated regions [37,38].

Another upstream enhancer element (downstream CNS0), located −4970 to −6021 in mouse DNA, comprises a CpG island with ~67% GC content. Its detailed analysis shows a sequence from −5786 to −5558, which includes 23 CpG, revealing that this region was highly demethylated in naturally occurring Tregs, while it was methylated in TGFβ-induced Tregs and conventional T cells (Tconv cells). The −5 kb upstream enhancer is also important in *Foxp3* induction in natural Tregs. In naïve CD4^+^CD25^−^ T cells, artificial demethylation by DNMTs inhibitor Aza enhances Foxp3 protein production, and can be boosted by addition of TGF-β to cell culture [8]. The aforementioned human homologue that includes 28 CpGs has different methylation levels between Tregs and Tconv cells, with substantial demethylation in the former. Furthermore, in patients with rheumatoid arthritis, some differences in the upstream enhancer region were observed, as well as in the level of DNMTs. Research has revealed that this region’s methylation was negatively correlated with *Foxp3* mRNA expression levels, and in vitro methylation diminishes its activity [34]. These observations were confirmed in another study including 8 CpGs located from −5835 to −5794. In addition to weakening *Foxp3* expression, Tregs with upstream enhancer methylation had impaired suppressive functions [39].

With reference to the upstream enhancer methylation pattern, the discrepancies between the regulatory T cells and other CD4+ cells that are capable of transient Foxp3 production can be revealed by the examination of the *Foxp3* promoter region. By comparing the different CpG sites in the human putative promoter region, it was shown that positions −113, −77, −65, and −58 were significantly diversely methylated between CD4^+^CD25^high^Foxp3^+^ and CD4^+^CD25^low^ cells. Moreover, the CpG site −77 was the most differently demethylated between these two populations. Therefore, it was considered as a pivotal region for cell differentiation. In reference to stable *Foxp3* expression, only Tregs that had almost complete promoter demethylation were committed to the Tregs population and remained functionally stable [40]. Another study on the methylation status of 10 CpG sites within the 451 bp region of *Foxp3* promoter has disclosed complete demethylation in Tregs and substantial methylation in CD4^+^ conventional cells [41]. In studies of patients with diseases that are connected with impaired Tregs function, such as biliary atresia, systemic sclerosis, fulminant type 1 diabetes (T1D), and recurrent spontaneous abortion, it was noted that the level of mRNA *Foxp3* was negatively correlated with higher promoter methylation. This relation might be responsible for diminished suppressive function of Tregs and further stand behind the pathogenesis of many diseases [42,43,44,45].

Furthermore, the DNA methylation pattern has been extensively studied at the *Foxp3* gene locus on several downstream intronic enhancers, described as CNSs: 0–3, according to their distance from the TSS.

CNS1, containing binding sites for SMAD and NFAT, is necessary for the induction of peripheral Tregs, and its depletion causes spontaneous abortion of embryos [36,46,47]. Peripheral Tregs generation in mice lacking the CNS1 region is attenuated, and leads to impairment of the Tregs-related mechanisms [48]. The importance of DNA methylation in this region was unleashed through the study on TET family proteins, where the loss of TETs led to diminished suppressor function of Tregs due to impairment of CNS1 demethylation [49]. Similar conclusions were reached in another study on a mouse model. It was shown that TET2 and TET3 were enzymes mediating CNS1 demethylation during cell development, particularly this TET-dependent demethylation occurred gradually in Tregs after the CD4 SP stage. Additionally, the study on 4 CpGs in CNS1 has shown that WT mice were highly unmethylated, while Tet2/3 DKO mice had substantial methylation (20–30%), which resulted in instability in cell culture and lower suppressor function in vitro [26].

Extensive research on induced Tregs has revealed their instability in cell culture and diminished suppressor function in vivo, which might be connected with the overall increase in CNS1 methylation. In order to improve their function, many substances are believed to upregulate their properties, e.g., vitamin C [50,51]. The addition of vitamin C in mouse, as well as human, iTregs caused CNS1/2 demethylation, leading to an increase in suppressor functions [26].

Furthermore, another locus—CNS2—is also under TET regulation [49]. In the research on 11 CpGs methylation in mice Tregs precursors, CD25^+^Foxp3^−^ and CD25^−^Foxp3^+^, it was confirmed that progressive CNS2 demethylation occurred in the latter [26]. This progression was disturbed when the TET2/3 enzymes were depleted from the cell precursors. In mice models, it was revealed that TET2/3-deficient-Tregs were less stable and more likely converted into Th17 cells, having higher methylation in the CNS2 locus [26,52].

CNS2 contains binding sites for the NFAT, c-Rel, CREV, Runx1, Ets-1, GATA3, and STAT3 transcription factors, ensuring stable expression of Foxp3. As the presence of each of these TFs is crucial, studies using specific knockdown models have shown that CNS2-related TFs deprivation did not affect Tregs maturation, but rather contributed to Tregs instability in cell culture due to decreased Foxp3 expression [53,54,55,56,57]. Therefore, DNA demethylation in the CNS2 region of the *Foxp3* gene is believed to be an important element ensuring Tregs stability and functionality and called the Major Treg-Specific Demethylated Region (TSDR). The importance of TSDR demethylation is indicated by instability of the in vitro-generated Tregs, which easily lose their phenotype and function due to methylation in CNS2. In addition, the previously mentioned TFs can bind to this region only when they are unmethylated, triggering Foxp3 expression [58,59]. From many TFs, the Runx1–Cbfβ–Foxp3 complex seems to be the most important. Runx1 and its cofactor Cbfβ together interact with demethylated CNS2 and allow continuous production of Foxp3 in a mechanism called a feed-forward loop. Moreover, DNA demethylation in TSDR provides ‘cell memory’ and ensures regulatory T cell stability upon pro-inflammatory conditions. Cells that have DNA demethylation in TSDR, and partly have lost Foxp3 expression under certain conditions, are able to restore Foxp3 expression due to epigenetic recollection [59,60]. It is believed that the TSDR methylation status, more than *Foxp3* expression, is a reliable factor that can predict the quality of regulatory T cells during cell culture because of the fact that the mRNA and protein levels are unstable parameters [61,62]. Likewise, TSDR demethylation is an exclusive marker of regulatory T cells that allows discriminating Tregs from activated effector cells. This is because T effectors may acquire transient Foxp3 expression upon activation [63]. Considering the Tregs populations, nTregs acquire stable and profound TSDR demethylation, while iTregs remain almost completely methylated, which makes them less stable during cell culture [64]. In addition, Tregs that are generated in vivo have similar, albeit lower TSDR demethylation compared to nTregs [65].

The proper methylation pattern in the CNS2 locus is promoted by Blimp1. This protein restricts DNMT3a from mediating the methylation of TSDR and consequently leads to cell stability [66]. Another protein—Mbd2—is considered as a factor that promotes DNA methylation; conversely, in Tregs it contributes to the maintenance of TSDR demethylation and its targeting is associated with impaired Tregs function [67]. A recent study by the Sakaguchi research group has revealed that stable Tregs could be generated from Tconv cells upon abrogation of the CD28 signaling pathway. This mechanism blocked the CD28–PKC–NF-kB axis and enabled demethylation in the CNS2 locus in Tregs generated in vitro, which made them stable and effective [68].

The last sequence that controls the *Foxp3* locus—CNS3—controls the tTregs and pTregs number. It was suggested that this region was not crucial for the maintenance of Foxp3 expression, rather than for early modification of the *Foxp3* locus. Thus, this pioneer element allows the poised state of the *Foxp3* promoter, enabling responsiveness to TCR stimuli [69]. Due to the fact that this region binds c-Rel, a TF highly important for *Foxp3* induction, it is believed that cooperation with c-Rel-CNS2-CNS3 enables recruitment of other TFs and eventually leads to stable demethylation of CNS2 in nTregs [36].

### 3.3. Epigenetic Modifiers of the Foxp3 Locus

Due to massive participation of DNA methylation in Tregs regulation and development, and the reversibility of epigenetic changes, there is a possibility to mediate Tregs function by epigenetic pharmaceutics, such as cytosine modifiers. DNMTs inhibitors (DNMTIs), such as azacytidine, decitabine, and zebularine, are a group of suitable candidates. The first two are approved by the FDA (Food and Drug Administration) for the treatment of hematologic disorders, but the last one, until now, has no approval [70,71]. Besides the impact of DNMTIs on cancer, their properties are used to modulate regulatory T cell function [8].

The DNA methyltransferase inhibitor azacytidine (AZA) is used in the treatment of AML (acute myeloid leukemia) or MDS (myelodysplastic syndromes) after allo-SCT (allogenic blood stem cell transplantation). The treatment results in diminished induction of graft-versus-host disease (GvHD) due to enhancement of Foxp3 expression and further Tregs expansion [72,73]. The profitable effect on 5-AzaC was observed in patients with MDS, in which the overall Tregs number increased because of CD25- cells’ conversion into regulatory T cells. Additionally, Tregs cultured with AZA had lower promoter methylation compared to the control [74]. Mouse research on an AZA derivative, decitabine, has led to similar conclusions, where naturally occurring Tregs played an essential role in protecting from GvHD after exposure to AZA [74,75]. Consistent with the data, treatment with a low dose of AZA augmented *Foxp3* RNA expression in CD4^+^Foxp3^−^ cells, and increased the total amount of CD4^+^CD25^+^ cells, leading to mitigated T effector cells activation. Altogether, these effects prevented experimental autoimmune encephalomyelitis [76]. Moreover, Tregs that have been modulated by azacytidine during inflammatory conditions in mice were highly effective, and TSDR demethylation in iTregs was about 80%, while iTregs without AZA had about 5% of TSDR demethylation. Given the fact that iTregs are highly unstable, the addition of AZA might be a promising tool for iTregs improvement [77].

Similar results were obtained by another research group, which have revealed a positive effect of another DNA methyltransferase inhibitor—decitabine (5-aza-2′-deoxycytidine)—on Tregs populations. They found a trend of diminished TSDR methylation in Tregs after decitabine (DAC) treatment. In mice models, it was established that DAC-treated Tconv cells have acquired the ability to produce Foxp3, mediated suppressor functions, and revealed strong expression of Tregs-related molecules [78,79]. Nevertheless, mice treated with DAC consisted of a higher percentage of Foxp3-positive cells in the thymus, with lower intron 1 and promoter methylation, which in turn prevented cyclophosphamide-induced T1D [79]. Although suppressor functions of DAC-treated effector cells were achieved in the mouse model, studies on human cells did not confirm this. Human CD4^+^CD25^−^ cells stimulated with DAC possessed only partial TSDR demethylation and were able to produce Tregs-specific genes but in smaller amounts compared to nTregs. Despite having some features of nTregs, DAC-induced Tregs did not have any functional activity [80]. In addition, patients infected with HBV and subjected to DAC treatment had higher percentages of Tregs and *Foxp3* [81].

On the other hand, zebularine (ZEB)—a less toxic reagent, which has significant demethylating properties—has not been yet approved by the FDA [82]. Similarly, research in mice models on azacitidine and decitabine function found that ZEB promoted *Foxp3* expression and CpGs demethylation of the Tregs’ upstream enhancer. In addition, human CD4^+^ ZEB-mediated cells had diminished INF-γ and IL-17A expression [83].

Furthermore, rheumatoid arthritis (RA) patients treated with methotrexate (MTX), which reduces the level of DNMT1, were characterized by restored Tregs functions and higher *Foxp3* expression caused by a decrease in upstream enhancer methylation [84].

The second group of epigenetic modifiers are factors that influence TET-mediated active DNA demethylation, such as vitamin C, which has a profound effect on iTregs. Vitamin C, a known coactivator for TET proteins, upregulates these proteins in iTregs. The stability and suppressive function of these cells are increased due to the lower methylation of CNS1 and CNS2 [26]. Despite the fact that pTregs are under vitamin-C influence, vitamin-C deprivation does not influence nTregs, because these cells have substantial TET expression, keeping CNS2 demethylated [85]. Low oxygen culture conditions, alone or in combination with vitamin C, can induced a stable phenotype in TET-transduced iTregs. These murine iTregs not only had demethylation in Treg-related genes but also were more suppressive compared to normal iTregs [27]. The research conducted on mice has shown a beneficial effect of stimulated iTregs on the prevention of GvHD or allograft rejection [86,87]. More complex research on iTregs, including the cooperation of three epigenetic modifiers, including vitamin C, has disclosed a deep CNS2 demethylation in stimulated cells with high stability and usefulness in therapeutic trials [64].

Another reagent—hydrogen sulfide (H2S), enables TET1/2 binding to promoter region, and its deficiency abrogates Tregs function. Similar to TET-deleted mouse Tregs, an insufficient H2S level results in high methylation of promoter and CNSs regions [88]. The inhibition of TET activity by 2-hydroxyglutarate can be resolved using aminooxyacetic acid (AOA). This reagent selectively shifts the balance towards iTregs, blocking Th17 differentiation, and consequently mitigates experimental autoimmune encephalomyelitis (EAE) in mice. Namely, AOA reduces *Foxp3* methylation in the promoter region in TH17 cells, simultaneously decreasing CNS2 methylation in iTregs and TH17 cells [89].

The indisputable effect of IL-2 on Tregs functionality is connected to the epigenetic signature. Namely, mice Tregs lacking IL-2 had diminished expression of TET and lower TSDR demethylation [90]. During cell expansion, IL-2 allows stable demethylation and preserves the function of the iTregs [91].

CRISPR–dCas9-based technology, which allows for the manipulation of the gene expression in a specific DNA locus, is a promising tool to target the *Foxp3* gene. Okada et al. conjugated guide RNA (gRNA) with a catalytic domain of TET1 and showed partial demethylation in CNS2 in primary T cells. Even so, they did not see stabilization of Foxp3 expression [92]. Similar data were obtained in another study, where the CRIPSR method caused TSDR demethylation with subsequent *Foxp3* expression but no typical Tregs properties were observed [93].

Moreover, other known factors can mediate alteration in the methylation status of the *Foxp3* gene. One of them—an active metabolite of vitamin A, ATRA—is connected with Tregs promotion by targeting methylation at the promoter of the *Foxp3* gene. CD4^+^ cells from patients with systemic sclerosis subjected to ATRA were more abundant in *Foxp3* at the mRNA and protein level. However, the mechanism of ATRA-mediated demethylation remains unknown [94]. On the other hand, this metabolite of vitamin A does not cause any differences in TSDR methylation in iTregs and thus is not able to enhance iTregs stability through DNA demethylation [12]. Although, enhancement of iTregs generation and stability through co-action of ATRA and TGF-β can be achieved by modulation of histone acetylation [95]. Similarly, Tregs stimulated with rapamycin (RAPA) alone or together with ATRA after expansion were more stable, with sustained TSDR demethylation and better suppressive function of these cells [62]. Nevertheless, RAPA alone or with TGF-β, failed to induce TSDR demethylation in iTregs in all 11 analyzed CpGs [96].

**Figure 2:**
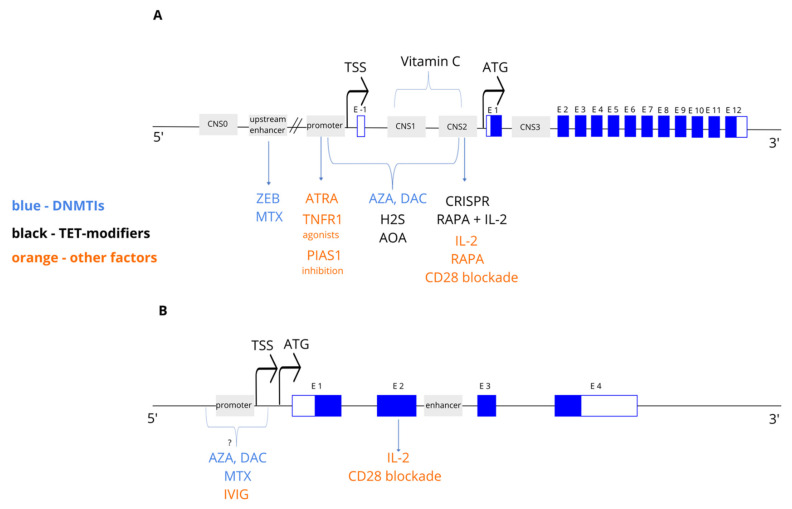
Overview of the structure of the *Foxp3* (**A**) and *Ctla4* (**B**) genes and the location of the epigenetic modifiers’ action. CNS0-1—conserved-non-coding elements; E—exon; TSS—transcription start site; ATG—translation initiation codon; ZEB—zebularine; AZA—azacytidine; DAC—decitabine; MTX—methotrexate; ATRA—all-trans retinoic acid; H2S—hydrogen sulfide; AOA—aminooxyacetic acid; RAPA—rapamycin; IVIG—intravenous immune globulin.

Looking into other manipulations, TNFR2 agonist or TNF1 antagonists can be used to enhance Tregs function by promoting demethylation in the proximal promoter of the *Foxp3* gene [97]. It can be also achieved through blocking of PIAS1 (SUMO E3 ligase), which recruits DNMTs and leads to chromatin repression of the *Foxp3* locus. The study on PIAS1-depleted mice has shown reduced methylation of the *Foxp3* promoter in developing CD4^+^CD25^−^ cells, which might be related with higher production of Foxp3^+^ cells from cell precursors [98]. An overview of the abovementioned is shown in Figure 2A.

## 4. *Ctla4* Gene

### 4.1. Ctla4 Gene Structure

*Ctla4* is located in the long arm of chromosome 2 (2q33.2) [99]. It has a 76% overall homology with the murine gene, and consists of 4 exons separated by 3 introns [100]. Its transcript is 1997 bp long and encodes a 223 aa protein. In Tconv cells, the gene is activated after cell stimulation, but in Tregs *Ctla4* expression is constant [101,102]. The human proximal promoter, located 5′ upstream from the UTR (untranslated region), comprises a pivotal regulatory sequence located from −200 to −330 bp. This sequence binds with a key transcription factor, NFAT, leading to *Ctla4* gene upregulation [103]. In the case of Tregs, the promoter of *Ctla4* is bound by the Foxp3:NFAT1 complex. The cooperation through stable *Foxp3* expression accelerates gene expression and stabilizes the *Ctla4* promoter [104]. Another transcription factor, *Foxp1*, binds the *Ctla4* promoter region and coordinates the binding of *Foxp3* to the promoter region [105]. The murine gene sequence that controls gene expression is located 335 bp upstream of the *Ctla4* sequence, with the key regulatory element placed between −238 and −167 from the transcription start site [101]. Using ChIP-Seq gene analysis of the H3K4me3 and H3K4me1 peaks, an enhancer element was revealed in the human *Ctla4* gene located in intron 2 [106]. Moreover, *Ctla4* SEs, illustrated by H3K27ac abundance, were found in Tregs and conventional cells [38]. Treg-specific SEs in the *Ctla4* locus were observed from the pre-tTreg (precursor) developmental stage [35].

### 4.2. Methylation of Ctla4

Consistent with the previous notion that CpG methylation affects gene expression, the pattern of DNA methylation in particular locations has an impact on cell functionality. During Tregs development, precursor cells subsequently acquire *Ctla4* demethylation, and this process can be enhanced by IL-2 [14].

A *Ctla4* promoter analysis in patients with colorectal cancer and melanoma has shown that CTLA-4 overexpression was caused by promoter hypomethylation. The mechanism behind this is the upregulation of TETs (TET1, TET2, and TET3) and reduced amount of DNMTs. The subsequent Tregs over-activity is used by tumor cells to escape from immune response, leading to a poorer prognosis. Thus, the examination of methylation status of promoter genes can be used as a predictive marker for cancer therapy [107,108]. Similar results have been observed in patients with breast cancer, in which enzymes that target DNA methylation were differentially expressed compared to the control [109]. Higher methylation status (33.98% vs. 19.81%) of the promoter gene, lower *Ctla4* mRNA, and increased DNTMs expression were detected in patients with myasthenia gravis (MG), causing a reduction in Tregs-related cytokines (TGF-β and IL-10) [110].

Contrary to this study, no differences have been observed in the level of DNMTs in RA patients, who possessed a higher promoter methylation pattern. However, the analysis of 10 CpGs in the promoter region has revealed the methylation discrepancies between RA patients and healthy individuals in two CpGs, −658 and −793, with statistical significance in the former. What is more, the researchers confirmed that Tregs had a similar demethylation pattern to T effector cells in the promoter region, with the exception of two CpGs that have been differentially methylated in RA patients. This research also revealed the mechanism by which NFAT2 binds to the −658 promoter region, only when this region is demethylated [30]. Similar data were obtained in the research on promoter methylation in MG patients where statistically significant hypermethylation occurred at −658 and −793 CpGs [111]. It confirms the inverse correlation between promoter methylation and gene expression.

Apart from the promoter, *Ctla4* exons have different patterns between the regulatory and Tconv cells. Exon 2 was considered as a Tregs-specific region defining cell commitment and stability upon activation. However, CD4^+^CD25^−^ cells cultured with polarized conditions towards iTregs did not acquire demethylation in this region [12,16]. Research on mouse models has shown that alloantigen-specific iTregs generated in the presence of DCs had Treg-specific epigenetic marks, including *Ctla4*, and were able to mediate cell suppression [112]. Consistent with this study, antigen stimulation led to sustained demethylation in both iTregs and pTregs [113]. In another study, naïve T cells were almost completely methylated in exon 2 but effector memory T cells were demethylated at around 28%, while Tregs were demethylated in 92% [114].

Apart from the above, similar to SEs in the *Foxp3* locus, the *Ctla4* gene comprises specific enhancer elements, enriched in the hypomethylated regions. These CpG regions include the SNPs responsible for autoimmune diseases, e.g., T1D [38].

### 4.3. Ctla4 Modifications through Changes in DNA Methylation

The previously mentioned AZA not only acts on TSDR methylation in the *Foxp3* locus but also contributes to stabilization of *Ctla4* gene expression, which lasts after cell expansion. Enhanced conversion of human CD4^+^CD25^−^ cells into iTregs by AZA increases CTLA-4 expression. However, the combination of azacytidine and low-dose panobinostat (HDACi) decreased the Tregs function and cell population size due to lower production of Foxp3 and CTLA-4 [115,116].

As mentioned, these epigenetic modifiers, e.g., AZA, are widely used in cancer treatment strategies [117,118]. Nevertheless, it is worth emphasizing that DNA hypomethylating agents (DHAs) can upregulate immune checkpoint molecules, such as PD-L1, PD-L2, and CTLA-4. The increased expression of CTLA-4 can be connected with poorer response to cancer treatment. For this reason, many clinical trials, which include AZA or DAC therapy, combine the therapy with CTLA-4 or PD-L1/2 inhibitors, in order to improve the outcome of the treatment [119,120,121]. Apart from AZA and DAC, another DHA—guadecitabine—increases the level of *Ctla4* in melanoma and hematological cancer cells [122]. MTX also increases *Ctla4* expression indirectly via its action on the *Foxp3* methylation level and DNMT1 reduction. Like in the case of DHAs, it was responsible for suppressor activity of the Treg cells [84]. Despite the unequivocal effect of DNMTIs on upregulation of CTLA-4 expression, there are no data revealing the methylation status of the *Ctla4* gene in DNMTI-treated cells. However, DNMTIs promote demethylation in *Foxp3* and the *Gitr* promoter, leading to increasing gene transcription; it is possible that the same mechanism targets the *Ctla4* locus.

Another study has revealed the positive effect of intravenous immunoglobulins (IVIG) on CTLA-4 function. Tregs that have been expanded by DCs in response to IVIG had restored their suppressor abilities. It was reported that IVIG therapy diminished methylation in two CpGs in the promoter of the *Ctla4* gene that were highly methylated in non-treated MG patients [111]. The properties of IVIG to change the methylation level were confirmed by another study. However, the mechanism underlying the effect of IVIG on CpGs is poorly understood [123].

As previously mentioned, deprivation of the CD28 costimulatory molecule in Tregs resulted in high demethylation in the *Ctla4* locus compared to cells that expressed CD28, which might be another therapeutic maneuver [68].

On the other side, no beneficial effect on the *Ctla4* demethylation signature was observed in cells treated with vitamin C or retinoid acid [12,27]. An overview of the above mentioned is shown in Figure 2B.

## 5. *Il2ra* Gene

### 5.1. Il2ra Gene Structure

The human *Il2ra* gene is encoded in chromosome 10 (10p15.1). The transcript is 3218 bp and creates a 272 aa protein. It contains 8 exons, from which 6 are coding, while exon 8 and the 5′ fragment from exon 1 are noncoding [124]. A pivotal enhancer element (positive regulatory region—PRRI) in the promoter region is located from −299 to −228 relevant to the TSS and binds NF-κB [125]. Additionally, another human enhancer element (PRRII), comparable to the murine gene, is located between nucleotides −137 and −64, and comprises binding sites for Elf-1 and HMG-I(Y), and is believed to have basal promoter activity [126]. Third enhancer element (PRRIII), located ~3.7 kb from the TSS (−3700 to −3703), binds the Elf-1, Stat5, and GATA proteins. Its equivalent in the murine gene is a sequence between −1376 and −1304 [127]. PRRIV, on the other hand, is located within intron 1 (+3389 to +3596), both in humans and mice, while PRRV binds SMAD and CREB and is placed ~7.6 kb 5′ from the TSS [128,129]. The last one—PRRVI—is placed ~8.5 kb 5′ from the initiation transcription site, and is connected with the response to CD28 stimulation [130]. There are also two negative regulatory elements (NRE1/2) placed between −401 to −367 and −341 to −308, respectively [131].

The SEs stretched between the body gene and the sequence upstream of the TSS in the Il2ra gene binds STAT5 after cell activation by IL-2. CRISPR–Cas9-mediated deletion of the STAT5-binding sites within *Il2ra* SEs contributed to the diminished STAT5 binding and consequently resulted in lower IL2RA protein expression in T cells after 4 days of IL-2 stimulation [132].

### 5.2. Il2ra Methylation

Naturally occurring Tregs compared to Tconv cells have substantial demethylation in intron 1a in the *Il2ra* gene. Moreover, this Tregs-enhancer element is demethylated in freshly isolated cells, as well as cells subjected to the expansion procedure [13]. Upon stimulation, this region undergoes progressive demethylation in Tconv cells, indicating that demethylation in the intronic element of *Il2ra* is not restricted to the Tregs population. Additionally, CD4^+^CD25^−^ stimulation with TGF-β also leads to changes in methylation in the intronic region [12].

In the study on *Il2ra* methylation, including 18 CpGs in three CD4^+^ subpopulations, the trend towards diminished methylation in Tregs compared to naïve and memory CD4^+^ cells was revealed. Moreover, the CpG methylation pattern did not differ between the T1D cells and control groups, and upon activation, the naïve T cells underwent demethylation in PRRVI and +3502 CpG [133]. Samples analysis of whole blood from patients with autoimmune thyroid diseases (AITDs), in this case Hashimoto thyroiditis or Graves’ disease, have shown general hypomethylation in the promoter region in the latter [134]. Similar results from PBMC-derived samples have shown hypomethylation of the *Il2ra* promoter in children with obesity-associated asthma, which could be connected with inflammation in these patients [135]. In another study, differences in the methylation pattern in PBMC-derived DNA samples between the study and control group could not be seen due to indiscernibility in the mixed cellular population [136]. Consistent with that, the analysis was extended to discrimination of five subsets of cells—T and B-lymphocytes, neutrophils, natural killer, and monocytes—using population-specific expression analysis (PSEA). Such an action revealed that T cells were the only cells with a hypomethylated promoter region in patients with multiple sclerosis (MS) compared to healthy individuals. Moreover, these cells had higher *Il2ra* expression than the control group, indicating a link between methylation in the promoter region and gene expression [136]. The methylation pattern in the proximal promoter in 6 CpGs, −459, −456, −373, −356, −272, and −241, varies between different subsets of immune cells. Moreover, some alterations of the level of methylation in particular CpGs occur. The least methylated CpGs are −241, −272, and −256, while the most are −456 and −459. However, −373 has an intermediate methylation level. What is more, Tregs compared to other cells had lower methylation in −459, −456, and −373 CpGs. The overall methylation did not alter between the T1D patients and control group, with the exception of −456 and −373, which were more methylated in the T1D group [137].

Like in the case of *Foxp3* and *Ctla4*, many DRs are found in SEs in the *Il2ra* gene, in which autoimmune-disease-associated SNPs (e.g., T1D and MS) are located.

### 5.3. Il2ra Methylation Modifications

A positive correlation between mRNA *Il2ra* and its protein CD25 was confirmed, which can be used to estimate the effect of methylating agents on their levels [138]. Previously mentioned dynamic demethylation in the *Il2ra* locus, which can be achieved through T cell activation or CD25^−^ cells’ stimulation with TGF-β, is a potent mechanism targeting cell functionality [12]. Consistent with this, AZA or DAC promotes conversion of CD25^−^ cells into CD25-expressing Tregs, which have a methylation pattern similar to nTregs; these reagents should mediate demethylation in the *Il2ra* locus [74,75,76,77]. The positive effect of other hypomethylating reagents, such as vitamin C, on the conversion of CD25 negative cells into positive cells was observed in humans and mice [139,140]. In humans, vitamin C enhances transformation of γδ T cells into Treg-like cells, leading to alterations in global DNA methylation, such as a decreased level in the *Il2ra* gene [140]. As Il-2 is a homeostatic regulator for Tregs, resulting in exaggeration of cell expansion, the therapy with a low dose of Il-2 is another factor affecting these cells [141,142,143]. From regulatory elements in the *IL2ra* gene, PPRIV—especially one CpG (+3502)—correlates with T cell activation. The higher level of IL-2 in supernatants of CD4^+^ cell cultures was connected with lower DNA methylation and exaggerated *Il2ra* gene expression [133]. In addition, mice treated with combination therapy using RAPA and IL-2 had an increased number of nTregs, with the highest peak on Day 28, when TSDR was profoundly demethylated [144]. Research with non-human primates receiving low dose IL-2 or engineered IL-2 molecules showed excessive Tregs proliferation. These cells after in vivo expansion maintained high demethylation in exon 2 of the *Ctla4* gene and *Foxp3*-TSDR [145]. However, another study revealed no impact of IL-2 deprivation on TSDR methylation after 36-h stimulation [146]. In opposition, therapies using anti-CD25 blockade, such as daclizumab, result in Tregs depletion. Nevertheless, the remaining cells comprised the Treg-specific epigenome and were not much altered after anti-CD25 monoclonal antibodies treatment [147].

## 6. *Tnfrsf18* Gene

### 6.1. Tnfrsf18 Gene Structure

The human *Tnfrsf18* gene on chromosome 1 (1p36.33) encodes a protein called GITR. The 1083 bp-long transcript codes a 241 aa protein, and consists of 5 exons, separated with 4 introns. The 5′ of exon 1 and 3′ of exon 5 are non-coding elements. The homology between the human and murine gene is around 55% [148,149]. Analysis of the *Tnfrsf18* promoter region has shown that *Foxp3* binds to this region between −227 and −19 from the TSS, leading to enhanced histone acetylation, and thus higher gene expression [150].

In addition, research on the mice gene has revealed an enhancer element that is responsible for NF-κB binding, located ~5 downstream of the promoter region [151].

### 6.2. Tnfrsf18 Methylation

As TNFRSF18 expression on Tregs is constitutive. It was revealed that after CD4^+^CD25^−^ cells activation, substantial amounts of GITR are present on the cell surface [152]. The analysis of two CpGs: −121 bp to +125 bp and in exon 4 in the human *Gitr* gene, has confirmed total methylation in Tconv due to activation of DNMT1. As expected, Tconv possessed lower *Gitr* mRNA compared to Tregs and higher binding of DNMT1 and MBD4 to the promoter region [153]. During Tregs thymic maturation, illustrated by the decrease in CD24, it was reported that these cells underwent successive demethylation in the *Gitr* locus, which can be upregulated by the presence of IL-2 [14]. Regarding Tregs-specific hypomethylation regions, exon 5 in *Tnfrst18* is considered as a pivotal region, which remains demethylated upon cell activation. In addition, upon iTregs generation with the use of TGF-β, no reduction in *Gitr* methylation was observed [12]. Moreover, pTregs, generated in the tumor microenvironment, had reduced suppressive function and were hypermethylated in the Treg-related loci, e.g., *Tnfrsf18* exon 5, compared to nTregs [154]. In an animal model study, generated antigen-specific iTregs were able to acquire Tregs-specific hypomethylation in *Gitr* in exon 5, and prevented skin-graft rejection [113].

### 6.3. Tnfrsf18 Modifications

*Gitr* promoter hypermethylation was observed in patients with multiple myeloma, resulting in tumor progression. However, the treatment with AZA restored *Gitr* expression due to promoter hypomethylation [155].

Aza upregulation of the Tregs function has also been connected with higher expression of GITR on the cell surface, which can be done by demethylation in this region [77]. Consistent with these data, DAC was the agent that upregulated GITR in CD4^+^CD25^+^ cells [78]. Furthermore, miRNA-mediated knockdown of DNMT1 in Tconv has contributed to demethylation in regions specific to Tregs, including *Tnfrsf18* exon 5, as well as other commonly hypomethylated regions, such as exon 1 and −700 [12].

Despite the positive role of TET retroviral induction in iTregs on *Foxp3* CNS2 and *Il2ra* demethylation, no differences were observed in the *Tnfrsf18* locus. The addition of vitamin C with the combination of a low-oxygen cell culture, did not cause *Tnfrsf18* demethylation, indicating that this region is resistant to such a modification [12,85]. In another study, on alloantigen-induced Tregs, *Gitr* expression was detected but the addition of vitamin C did not alter the methylation level of this gene [86]. Another study revealed a little demethylation due to vitamin C. Nevertheless, it was not so profound as in other genes [86,87].

## 7. *Ikzf2* and *Ikzf4* Overview

Both *Ikzf2* and *Ikzf4* belong to the Ikaros family zinc finger protein 2. Based on the Ensembl database, the *Ikzf2* gene encodes a Helios protein and is located in chromosome 2 (2q34). Its 3888 bp transcript contains 9 exons, from which 7 are coding and encodes a 526 aa-long protein. *Ikzf4* encodes an Eos protein. It is placed on chromosome 12 (12q13.2). A 5314 bp transcript consists of 8 coding exons and creates a 585 aa-long protein. These two genes comprise a specific methylation pattern, which differs between nTreg and Tconv cells. Bisulfite sequencing of the *Ikzf2* gene revealed substantial demethylation in intron 3a and exon 6 in nTregs compared to Tconv cells, and a specific Tregs demethylated region in intron 1b of the *Ikzf4* gene. Moreover, in vitro-generated Tregs with or without retinoic acid or TGF-β and Foxp3-transduced Tconv cells do not acquire demethylation in *Ikzf2/4* genes (12). In addition, TGF-β deficiency in the environment of tumor-infiltrating cells did not much alter the methylation signature, including the *Ikzf4* region [156]. The discrepancies between the Tregs population were observed in another study, where pTregs and tTregs had a different methylation percentage of *Ikzf2* (complete methylation in the former) [157]. *Ikzf2* and *Ikzf4,* similar to the other abovementioned genes, also contain SEs, which are enriched in DRs. Regarding *Ikzf4*, these DRs are enriched in SNPs connected with autoimmune diseases. Development of Foxp3^−^ thymic precursor cells is impaired in Satb1-deprived mice, which is indicated by the lack of demethylation in Tregs-related genes, e.g., *Ikzf2* and *Ikzf4* [35,38]. Consistent with the previously mentioned role of TET proteins in the demethylation process, this enzyme is highly important in demethylation in intron 1b of the *Ikzf4* gene. The analysis of 6 CpG sites in mice lacking TET2 and TET3 proteins has revealed higher methylation in all examined CpGs compared to wild-type mice [49].

Despite the lack of influence of retinoic acid or vitamin C in the demethylation of the *Ikzf2* gene, the beneficial effect of the BCG vaccine on *Ikzf2/4* demethylation was confirmed in a study on T1D. Significantly higher demethylation in 8 out of 11 CpGs, due to the BCG treatment, was found in Tregs-related genes [27,158].

Moreover, CD28 blockade is another approach that can be used to manipulate the methylation status of these genes. It was shown that CD28^−^ iTregs had substantial demethylation in intron 1 of the *Ikzf4* gene and intron 3a of the *Ikzf2* locus [68].

## 8. Conclusions and Future in Tregs Applications

Therapies using Tregs have been extensively developed over the last years. Tregs’ suppressive potential was used in the treatment of GvHD after bone marrow transplantation [159], in solid-organ transplantation [160], and in autoimmune diseases, such as T1D or MS [161].

In current clinical trials, polyclonal Tregs are utilized, whose positive effect and safety has been confirmed [162]. Polyclonal Tregs possess specificity towards multiple antigens, which decreases their tissue-specific effectiveness. For this reason, researchers are searching for a method to generate Tregs that would migrate precisely to the site of ongoing infection. Nowadays, several methods for generating antigen-specific (Ag-spec) Tregs are known.

Tang et at. in their study on Ag-spec Tregs in transgenic mice have shown higher suppressive capacity of these cells compared to polyclonal Tregs. They have also indicated better migration properties and duration in the target tissue [163]. Another approach to generate specific Tregs is based on in vitro expansion with antigen-presenting cells (APCs) derived from donors. It has been found that the exposure to alloantigens presented by DCs of B cells resulted in specific Tregs expansion [164,165]. Moreover, our research team has developed a method that uses monocytes presenting antigens to create Ag-specific Tregs. We have shown that Ag-spec Tregs were stable during cell culture and possessed higher suppressive properties than polyclonal cells [166,167]. Other approaches use transfection of viral vectors encoding TCRs or chimeric antigen receptors (CARs). In murine models, tumor-specific Tregs generated by lentiviral transfer of TCRs suppressed tumor-specific Teffs, which was indicated as tumour growth [168]. In reference to retroviral CAR transfection, lung epithelial-directed Tregs were able to diminish the immune response in a murine airway hyper-reactivity model [169]. The CRISPR–Cas9 method, based on gene knock-in and knock-out, is another that may improve the therapeutic effects of the cells via upregulation of the genes crucial for Tregs [170,171].

These methods are believed to be a potential tool for providing effective and safe Tregs therapies. In addition, the previously mentioned substances, the so-called epigenetic modifiers, could be another factor boosting Tregs function (Table 1).

The possibility to create mixed protocols to create antigen-specific Tregs, with the addition of epigenetic modifiers, is a promising idea for future clinical trials. However, it is crucial to remember that these substances drive changes in non-Tregs too. For this reason, not only efficacy but also safety studies should be performed before wider clinical applications.

## Figures and Tables

**Table 1:** The discussed factors that can mediate changes in the DNA methylation pattern are listed with the mechanism of action and the direct role on the function of regulatory T cells.

Epigenetic Modifier	Affected Genes	Direct Effect on Gene	Direct Effect on Tregs	Reference
Vitamin C	*Foxp3, Ctla4, Il2ra, Ikzf4*	-increased CNS1/2 demethylation in iTregs in *Foxp3* gene -decreased methylation of Tregs DMRs in all genes	-enhancement of iTregs stability during cell culture -boosting *Foxp3* mRNA production -improving iTregs function and usefulness in therapies	[27,64,86,87]
IL-2	*Foxp3*, *Ctla4*, *Tnfrsf18*, *Ikzf4*	-decreased methylation of Tregs DMRs	-induction of demethylation in Treg precursors -preserving cell stability upon expansion	[43]
H2S	*Foxp3*	-higher promoter demethylation	-maintenance of Tregs properties	[88]
AOA	*Foxp3*	-increasing demethylation of promoter and CNSs	-promotes Treg-like polarization	[89]
Vitamin A	*Foxp3*	-demethylation in promoter of *Foxp3*	-increasement of mRNA and protein level of Foxp3 -enhanced pTregs production	[94]
MTX	*Foxp3, Ctla4*	-lower upstream enhancer methylation in *Foxp3* gene	-higher Foxp3 expression in cells from RA patients -higher CTLA-4 expression	[84]
RAPA	*Foxp3*	-stabilizes TSDR demethylation upon expansion	-protect Tregs phenotype and functional stability during cell expansion	[62]
AZA DEC ZEB	*Foxp3, Ctla4*, *Tnfrsf18*	-decreasing promoter and TSDR demethylation in *Foxp3* gene -lower methylation of promoter in GITR gene	-increasing overall pTregs number -conversion of CD4+CD25- cells into Tregs -enhanced Foxp3, CTLA-4 and GITR expression	[35,74,75,76,77,78,79,80,83,116,117,119,120,121,122]
CD28 signaling pathway blockade	*Foxp3, Ctla4, Ikzf2/4*	-decreased methylation of Tregs DMRs in all genes	-generation of stable iTregs having comparable epigenome to nTregs	[68]
TNF1 antagonists	*Foxp3*	-lower promoter methylation	-increasing Tregs stability upon inflammatory environment	[97]
PIAS1 deletion	*Foxp3*	-lower promoter methylation	Higher production of Foxp3+ cells	[98]
CRISPR-dCas9-TET1CD	*Foxp3*	-induction of TSDR demethylation	-Foxp3 production in primary human T cells	[92,93]
IVIG	*Ctla4*	-demethylation of promoter	-upregulation of CTLA-4 transcript and protein level	[111]
BCG	*Foxp3, Il2ra, Tnfrsf18, Ikzf2, ikzf4*	-reduction of DNA methylation at multiple CpGs	-higher production of mRNA	[158]

ZEB—zebularine; AZA—azacytidine; DAC—decitabine; MTX—methotrexate; ATRA—all-trans retinoic acid; H2S—hydrogen sulfide; AOA—aminooxyacetic acid; RAPA—rapamycin; IVIG—intravenous immune globulin.

## Data Availability

Not applicable.
